# Global Characterization of Metabolic Genes Regulating Survival and Immune Infiltration in Osteosarcoma

**DOI:** 10.3389/fgene.2021.814843

**Published:** 2022-01-13

**Authors:** Zhongpei Zhu, Min Zhang, Weidong Wang, Peng Zhang, Yuqiang Wang, Limin Wang

**Affiliations:** ^1^ Department of Orthopedics, The First Affiliated Hospital of Zhengzhou University, Zhengzhou, China; ^2^ Department of Orthopedics, Tumor Hospital of Henan Province, Zhengzhou, China

**Keywords:** osteosarcoma, immune infiltration, metabolic genes, differentially expressed genes, PAICS

## Abstract

**Background:** The alterations in metabolic profile of tumors have been identified as one of the prognostic hallmarks of cancers, including osteosarcoma. These alterations are majorly controlled by groups of metabolically active genes. However, the regulation of metabolic gene signatures in tumor microenvironment of osteosarcoma has not been well explained.

**Objectives:** Thus, we investigated the sets of previously published metabolic genes in osteosarcoma patients and normal samples.

**Methods:** We applied computational techniques to identify metabolic genes involved in the immune function of tumor microenvironment (TME) and survival and prognosis of the osteosarcoma patients. Potential candidate gene *PAICS* (*phosphoribosyl aminoimidazole carboxylase, phosphoribosyl aminoimidazole succino carboxamide synthetase*) was chosen for further studies in osteosarcoma cell lines for its role in cell proliferation, migration and apoptosis.

**Results:** Our analyses identified a list of metabolic genes differentially expressed in osteosarcoma tissues. Next, we scrutinized the list of genes correlated with survival and immune cells, followed by clustering osteosarcoma patients into three categories: C1, C2, and C3. These analyses led us to choose *PAICS* as potential candidate gene as its expression showed association with poor survival and negative correlation with the immune cells. Furthermore, we established that loss of *PAICS* induced apoptosis and inhibited proliferation, migration, and wound healing in HOS and MG-63 cell lines. Finally, the results were supported by constructing and validating a prediction model for prognosis of the osteosarcoma patients.

**Conclusion:** Here, we conclude that metabolic genes specifically *PAICS* play an integral role in the immune cell infiltration in osteosarcoma TME, as well as cancer development and metastasis.

## Introduction

Osteosarcoma is a rare type of cancer that affects the individuals of all ages including children (Yang et al., 2021). However, because of the advancements in chemotherapy, precise radiotherapy, and immunotherapy, the overall 5-year overall survival (OS) rate of patients has substantially increased to 70% (Li et al., 2021). It is noteworthy that only 15% to 20% of the patients were usually diagnosed with metastases, and the OS of these patients was reported to be extremely poor (Durnali et al., 2013; Zhang et al., 2019). Recently, immunotherapy, such as adoptive cellular therapy, vaccination, and checkpoint inhibitors, has been considered as the effective therapies for osteosarcoma (Lettieri et al., 2016; Heymann et al., 2019). Therefore, it is worth studying the underlying molecular mechanisms of occurrence and development of osteosarcoma with a deep focus on the identification of novel diagnostic, therapeutic, and prognostic markers (Yiqi et al., 2020).

In recent years, much attention has been paid to the contents of the tumor microenvironment (TME) and their roles in cancer development. It has been found that TME plays a critical role in the cancer development and recurrence (Ren et al., 2021). In short, the TME contains a variety of cells, including tumor cells, fibroblasts, endothelial cells, immune cells, various signal molecules, and extracellular matrix (Cortini et al., 2016; Chen et al., 2020; Luo et al., 2020). The increasing evidence shows that infiltrating immune cells such as T cells, B cells, macrophages, dendritic cells, monocytes, neutrophils, and mast cells may be involved in cancer development and progression (Du et al., 2021; Gerard et al., 2021; Kemmerer et al., 2021). In TME, tumor cells can invade surrounding tissues or metastasize through lymphatic vessels, and the infiltrated cells can stimulate host immune response for releasing cytokines, cytokine receptors, and other factors, which directly or indirectly promote or inhibit tumor cell proliferation (Federico et al., 2021; Zhang et al., 2021). The osteosarcoma TME is now considered as an essential element of tumor growth and dissemination (Corre et al., 2020). A latest study has shown the association of the tumor immune cell infiltration with clinical outcomes of osteosarcoma patients (Chen et al., 2020). Extensive studies on the TME have shown that infiltrating immune cells play a vital role in tumor growth, recurrence, metastasis, and response to the immunotherapy (Ma et al., 2020; Zhang et al., 2020). However, the detailed profile of immune cells infiltrating in osteosarcoma TME has not been elucidated yet (Zhang et al., 2020).

Previous studies have primarily focused on the one or two kinds of immune cells or key genes, which could bias the osteosarcoma microenvironment exploration. The identification of multiple genes from tumor-infiltrating immune cell profiles can help to construct a gene signature with better and more accurate prognostic potential. So far, no study has explained the role of metabolic genes in immune cell infiltration osteosarcoma TME and cancer development. Here, for the first time, we collected raw RNA-seq data from two different studies (Haider et al., 2016; Peng et al., 2018) and identified the top 10 differentially expressed metabolic genes in osteosarcoma tumors. Furthermore, we performed an *in silico* analysis to find their roles in osteosarcoma tumor progression. We also studied the role of these genes in immune cell infiltration in osteosarcoma TME, and a prediction model was also developed to predict the OS and prognosis of the osteosarcoma patients.

## Materials and Methods

### Clinical Samples and Data Acquisition

The RNA sequencing data of osteosarcoma samples and normal tissues were downloaded from two previously published studies (Haider et al., 2016; Peng et al., 2018). Complete clinical information (age, sex, primary tumor site, metastatic state at diagnosis, survival time, and survival state) were also included in our study. We included only genes expressing more than 50% of the samples and had expression level >1. The detailed workflow of the study is shown in Supplementary Figure S1.

### DEGs and Enrichment Analysis

The DEGs were identified by the difference of logFC in the expression between tumor and normal samples, genes having logFC >1 were considered for analysis. All genes were analyzed using R package limma. The differences in expression of the genes were presented as heat map. The selected genes were analyzed for the molecular, biological, and cellular functions by Kyoto Encyclopedia of Genes and Genomes (KEGG) and Gene Ontology (GO) enrichment programs.

### Construction and Validation of Prediction Model

The univariate and multivariate Cox HR regression analyses were performed to estimate the risk score and clinical features of the osteosarcoma patients. The risk score and clinical factors were used to identify the independent risk factors associated with the OS. The area under the receiver operating characteristic (ROC) curve (AUC) was calculated by using survival ROC R package to validate the performance of the prediction model. The 95% confidence intervals for AUCs were obtained using the nonparametric bootstrap via survAccuracy Measures package. For an internal validation, the calibration curve was plotted to assess the performance of the prediction model for prognosis and survival of the osteosarcoma patients.

### Analyses of Immune Cell Infiltration in TME

The R package xCELL was used to explore the immune cell infiltration in osteosarcoma TME. Furthermore, we used ESTIMATE to analyze the purity of the osteosarcoma tumors. Based on the metabolic transcriptional profiles of the patients, the stromal and immune scores of the osteosarcoma tissues were estimated. The association among the infiltrating immune cells was also studied, and *p* < 0.05 was set as cutoff for analysis.

### Survival Analysis

The DEGs in osteosarcoma patients were studied for OS analysis. The univariate and multivariate Cox regression analyses were performed using R package. The Kaplan–Meier survival curves were plotted, and ggplot2 was used to visualize the data. The variables associated with the survival were screened out using LASSO (Ichikawa and Brenner, 1981); variables having a regression coefficient more than zero were selected for analyses.

### Cell Culture

The human renal cancer cell lines HOS and MG-63 were purchased from the American Type Culture Collection (Manassas, VA, USA) and cultured in modified eagle medium (SH30244; Hyclone, Logan, UT, USA) supplemented with 10% fetal bovine serum (FBS; 10,099,141; Invitrogen, Carlsbad, CA, USA) at 37°C and 5% CO_2_ in a humidified incubator.

### Real-Time Quantitative Polymerase Chain Reaction

The osteosarcoma cells were first lysed with TRIzol (9,109; TaKaRa, Tokyo, Japan), then whole RNA was extracted using standard chloroform/isopropanol method. The RNA samples were quantified by using NanoDrop, and cDNA was synthesized by using reverse transcriptase enzyme (205,311; Qiagen, Duesseldorf, Germany). Relative expression of genes was studied by performing real-time polymerase chain reaction (PCR) using Quantinova Syber green PCR kit (208,054; Qiagen). GAPDH was used as internal control. Primer sequences are listed in the Supplementary Table S1.

### Cell Function Assays

*PAICS* (*phosphoribosyl aminoimidazole carboxylase, phosphoribosyl aminoimidazole succino carboxamide synthetase*) was knocked down using siRNAs in osteosarcoma cells HOS and MG-63. The PAICS siRNAs and negative control were synthesized by GenePharma Corporation (Shanghai, China). The sequences are provided in Supplementary Table S1. To assess the cells’ viability, after 24 h of transfection, 1,000 cells were seeded into each well of the 96-well plates. Cell Counting Kit-8 (CK04; Dojindo Laboratories, Kumamoto, Japan) was used to examine the proliferation capacity of the cells at 0, 24, 48, and 72 h after seeding cells. Absorbance was measured at 450 nm. Standard Transwell columns were used to study the effect of *PAICS* knockdown on cell migration; an equal number of cells (NC and si-PAICS) were seeded into in upper chamber of the Transwell column containing FBS less media; the lower chamber of the column was supplied with media supplemented with 10% FBS. The migration of cells from the upper column to the lower column was observed after 24 h by simply blocking with 4% paraformaldehyde (PFA) and staining with 0.5% crystal violet, visualized, and photographed by inverted microscope fitted with Nikon camera. For apoptosis, 72 h after transfecting cells with siRNA, cells were treated with an apoptosis detection kit (KGA1013; KeyGEN BioTECH, Nanjing, China) and analyzed by flow cytometry (FACScan; BD Biosciences). The wound healing assay was performed by creating a monolayer at 90% confluence of cells after transfecting with siRNA in six-well plates. The difference in the wound recovery was photographed and measured for 24 h. The colony formation assay was performed by seeding cells transfected with siRNA in six-well plates (1,000 cells/well) and cultured for 10 days. Then, cells were fixed with 4% PFA and stained with 0.1% crystal violet; the colonies were photographed and counted for determining differences between NC and knockdown cells.

### Statistical Analysis

The statistical analyses were performed by using SPSS 25.0 (IBM, Inc., Chicago, IL, USA), GraphPad Prism 7 (GraphPad Software, San Diego, CA, USA), and R packages (version 3.6.4). The LASSO regression analyses were performed by using glmnet R package. The comparison of the immune cell infiltration between tumor and normal tissues was analyzed using Wilcoxon test. However, the Cox proportional hazards model by Survival R package and log-rank test were used to calculate the *p* value for survival analyses. *p* < 0.05 was considered as significant, and all experiments were performed in triplicate.

## Results

### Identification of Metabolic Genes With OS

**FIGURE 1:**
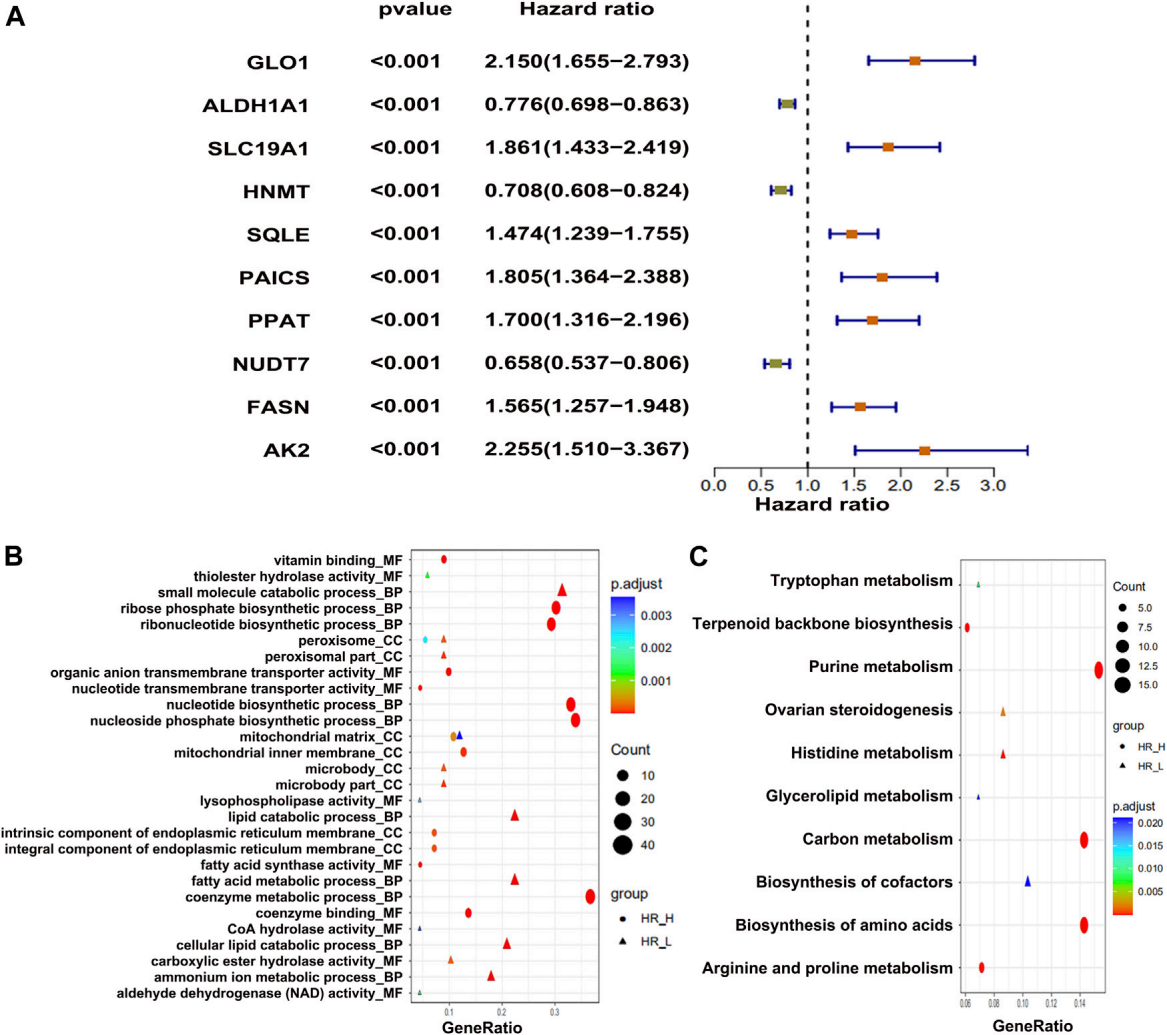
The differentially expressed metabolic genes in osteosarcoma tumors. **(A)** Top 10 metabolic genes, showing positive and negative association with survival. **(B)** GO enrichment and **(C)** KEGG enrichment analysis of top metabolic genes.

To identify the metabolic genes correlated with OS in osteosarcoma patients, we first downloaded the raw RNA-seq data of osteosarcoma patients mentioned in two different studies (Haider et al., 2016; Peng et al., 2018). We intersected only metabolic genes with expression greater than 1 and have 50% coverage across cancer tissues. The overall flowchart of the study is shown in Supplementary Figure S1. In total, we identified 876 metabolic genes. Next, we classified the top 10 metabolic genes associated with survival (Figure 1A). Among the top 10 genes, 3 metabolic genes (*ALDH1A1*, *HNMT*, and *NUDT7*) were negatively correlated, and 7 (*GL O 1*, *SLC19A1*, *SQLE*, *PAICS*, *PPAT*, *FASN*, *AK2*) were positively correlated with the OS of the patients (Figure 1A). Afterward, we performed GO (Figure 1B) and KEGG (Figure 1C) enrichment analyses for all survival-related metabolic genes. We also constructed a protein–protein interaction (PPI) network for these metabolic genes associated with survival (Supplementary Figure S2A). Lastly, we isolated two subgroups across the top 10 hub survival-related metabolic genes that were interconnected with each other (Supplementary Figure S2B). Both GO and KEGG enrichment revealed nucleoside biosynthetic process and purine metabolism as top enrichment terms, respectively.

### Identification of Metabolic Genes Correlated With CD8^+^ T Cells

**FIGURE 2:**
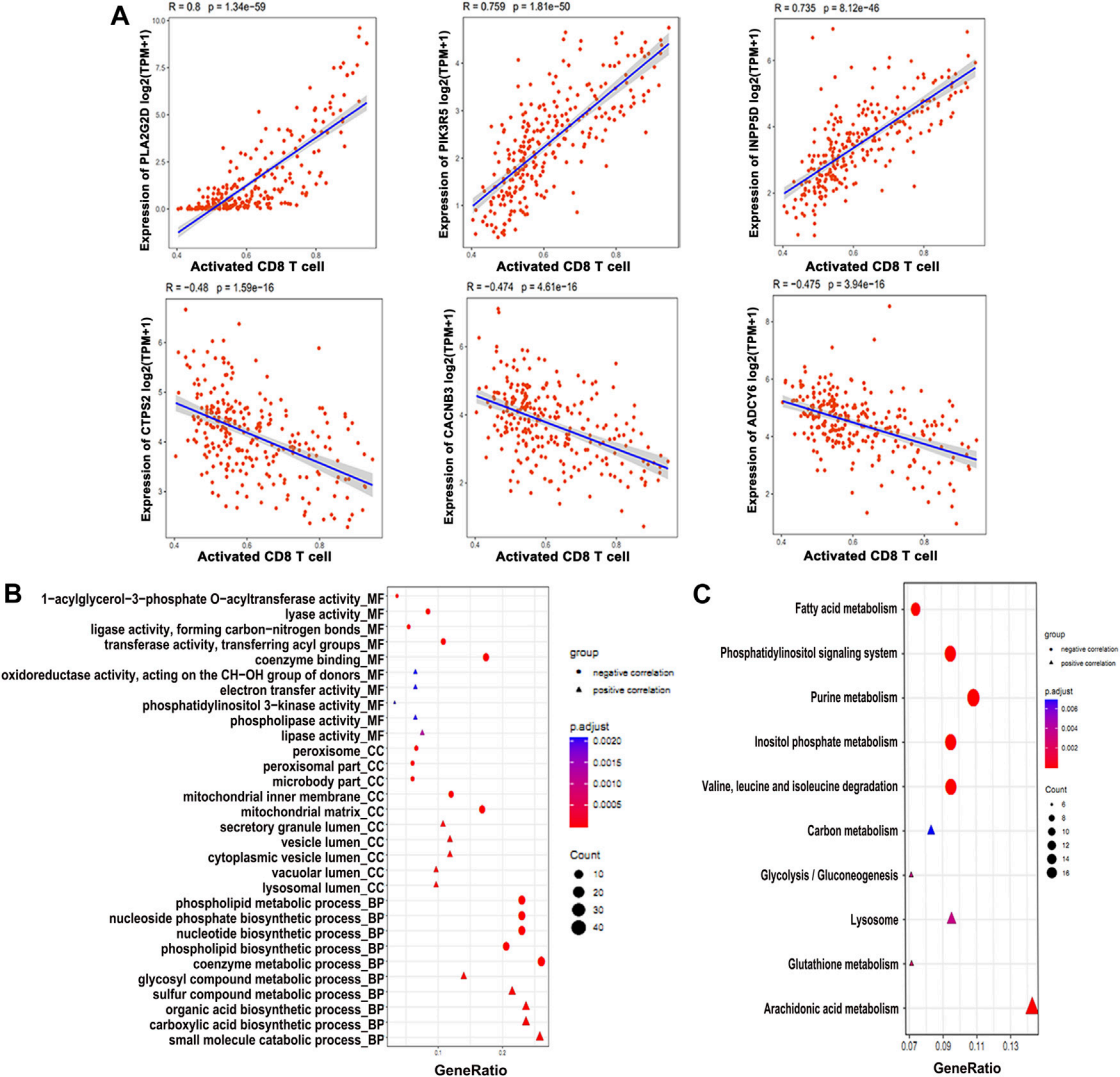
Differentially expressed metabolic genes involved in the immune function. **(A)** Expression profiles of the genes positively and negatively associated with CD8^+^ T cell. **(B)** GO enrichment and **(C)** KEGG analysis of the genes associated with the CD8^+^ T cell function.

We analyzed all metabolic genes to find correlation with immune cells, especially activated CD8^+^ T cells, which identified 206 differential metabolic genes correlated to immune cells. However, we found *PLAG2D*, *PIK3R5*, and *INPP5D* as the top three positively associated metabolic genes, with *R* > 0.7 (Figure 2A). On the other hand the *CTPS2*, *CACNB3*, and *ADCY6* were the top three negatively correlated metabolic genes with *R* < −0.50 (Figure 2A). GO enrichment analysis found that small_molecule_catabolic _process is one of the top BP terms associated with positively correlated metabolic genes (Figure 2B). On the contrary, the metabolic process of coenzymes was found to be enriched with negatively correlated metabolic genes (Figure 2B). KEGG pathway enrichment identified purine metabolism as the top pathway for negatively correlated genes (Figure 2C). Alternatively, arachidonic acid metabolism was positively correlated (Figure 2C). Next, we constructed PPI network for immune cell–related metabolic genes (Supplementary Figure S3A) and identified the top 10 hub immune-related genes (Supplementary Figure S3B). In conclusion, a total of 206 metabolic genes showed correlation with CD8^+^ T immune cells in osteosarcoma patients.

### Clustering Osteosarcoma Patients

**FIGURE 3:**
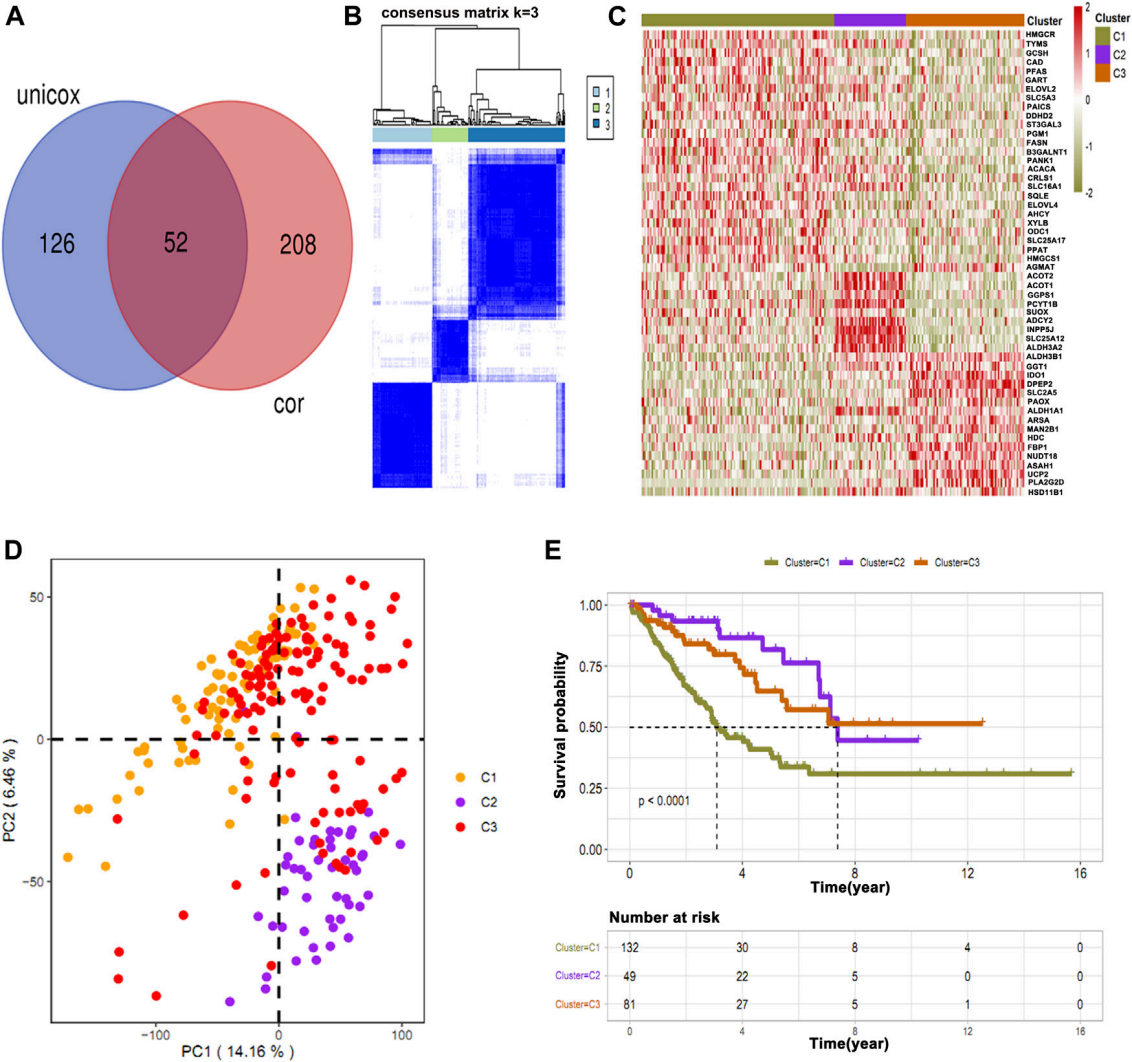
Intersection of common immune-related genes and clustering of osteosarcoma patients. **(A)** Unicox analysis showing 52 common immune-related genes. **(B)** Patients were clustered based on common genes. **(C)** Heat map showing the expression of immune-survival related genes in different clusters. **(D)** PCA showing high variance in C2. **(E)** Survival analysis in three clusters showing C1 has a poor survival rate as compared to other clusters.

Of a total 876 metabolic genes associated with OS and 206 associated with immune cells, we intersected common genes (Figure 3A). These common genes were then subjected to cluster analysis, and tumors were divided into three clusters: C1, C2, and C3 (Figure 3B). Subsequently, the heat map shows the expression of immune-survival related genes (Figure 3C). We observed differential expression of immune-survival related genes in three clusters (Figure 3C). The high variance between the three clusters was confirmed by principal component analysis (PCA) (Figure 3D). Furthermore, clusters C1 and C3 were closely grouped and compared with C2 in the PCA map of osteosarcoma. Lastly, in cluster grouping, we performed survival analyses and found that among the three clusters, C1 has the worst OS (Figure 3E).

### Immune Status of the Clusters

**FIGURE 4:**
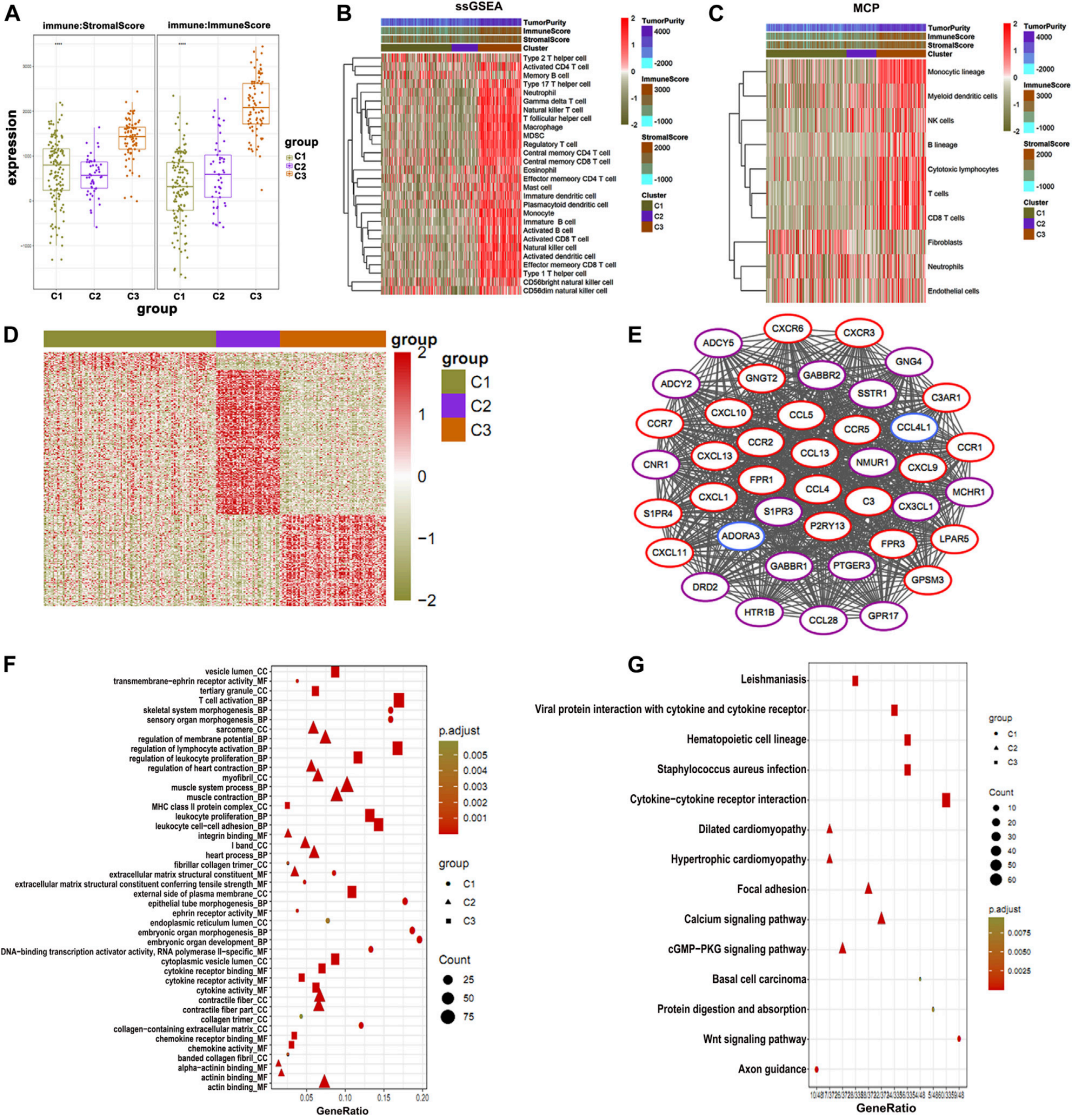
Immune status of osteosarcoma clusters. **(A)** C1 shows the lowest immune and stromal score than the clusters. **(B)** The ssGSEA and **(C)** MCP-counter analysis plotted in the form of a hierarchical heat map (C3) show the highest immune score. **(D)** Highly expressed genes in three clusters. **(E)** PPI of highly expressed genes showing association with immune function. **(F)** GO enrichment and **(G)** KEGG enrichment analysis of the genes associated with immune function.

To further identify the potential prognostic features for osteosarcoma, the stromal immune scores of metabolic gene signatures in clusters were determined (Figure 4A). The immune score in C1 was significantly low, whereas C3 had the highest immune score (Figure 4A). Next, we determined the level of immune infiltration and types of the immune cells in all osteosarcoma clusters using ssGSEA and MCP-counter, respectively. Both methods are considered as reliable computational methods for estimating the infiltration of immune cells in TME (Barbie et al., 2009; Becht et al., 2016). The data obtained from ssGSEA and MCP-counter analysis were plotted in the form of a hierarchical heat map (Figures 4B,C). As expected, C3 showed the highest level of immune infiltration as compared to C1 and C2 clusters. Immune infiltration provided a clear picture of differential patterns of the cells among three clusters. Furthermore, we analyzed differential patterns of the genes in all osteosarcoma clusters and determined highly expressed differential genes in each cluster. The close observation of each cluster revealed that only C3 was enriched with the high expression of genes associated with immune cells (Figure 4D). Moreover, we illustrated an interacting map of highly expressed genes in three clusters via hub gene diagram (Figure 4E). Finally, we performed GO and KEGG pathway enrichment of highly expressed genes in all clusters (Figures 4F,G).

### Phosphoribosyl Aminoimidazole Succino Carboxamide Synthetase Contributes to Poor Survival in Osteosarcoma Patients

**FIGURE 5:**
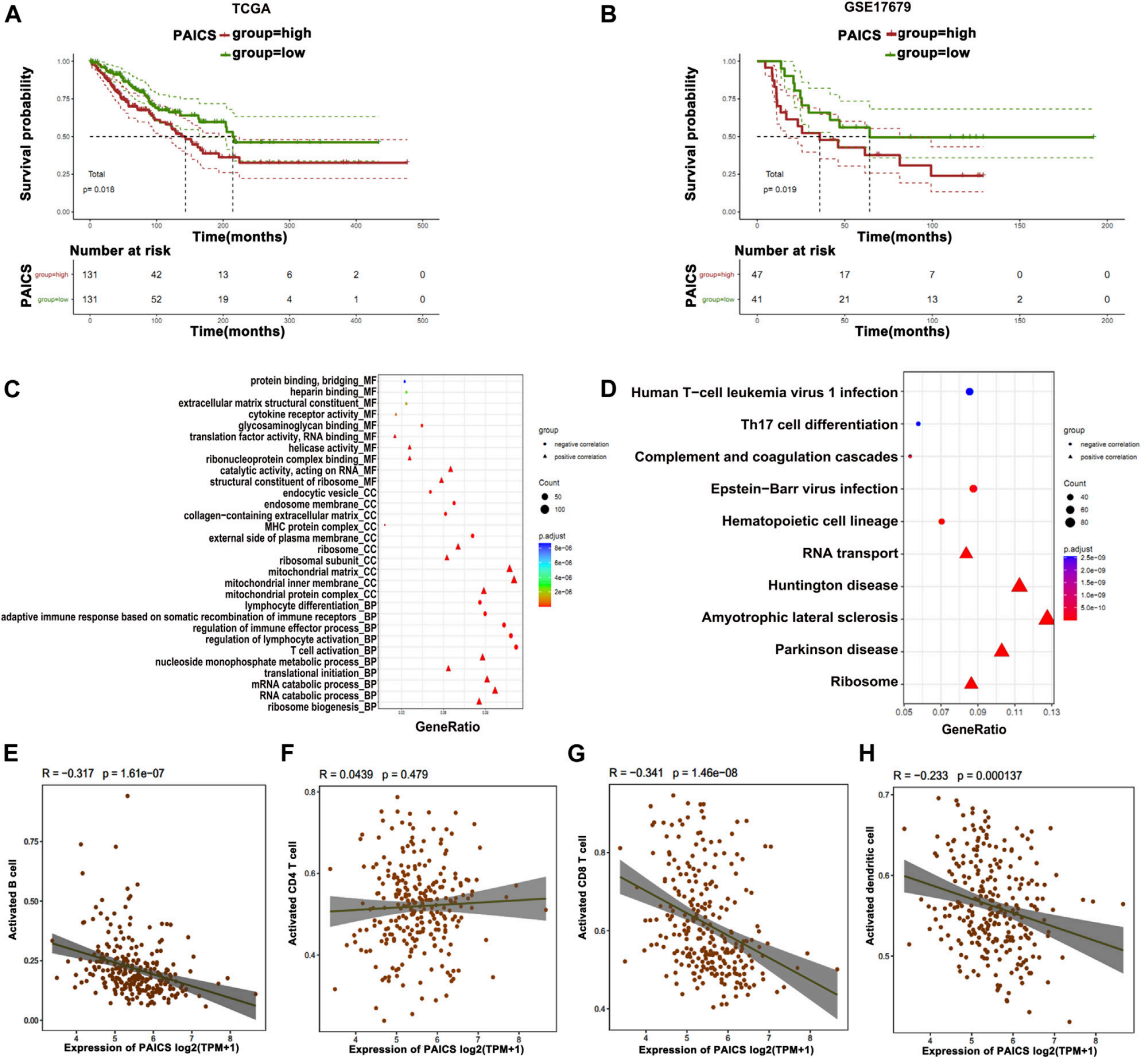
Prognostic features of *PAICS*. **(A**, **B)** TCGA and GSE17679 databases show overexpression of *PAICS* associated with poor survival. **(C**, **D)** GO and KEGG enrichment analysis of *PAICS* gene showing its role in immune cells’ function. Dot plots showing association of *PAICS* with **(E)** activated B cells, **(F)** activated CD4^+^ T cells, **(G)** activated CD8^+^ T cells, and **(H)** activated dendritic cells.

Among the top 8 metabolic genes, *PAICS* attracted our attention as it is known to be significantly involved in the *de novo* purine biosynthesis and tumorigenesis (Huang et al., 2020). The expression analysis of *PAICS* in several cancers (The Cancer Genome Atlas [TCGA]) showed high expression of *PAICS* in most of the cancers including osteosarcoma (SARC) (Supplementary Figure S4). Survival analysis showed that patients with high expression of *PAICS* had poor survival rate, and *vice versa* (Figures 5A,B). Next, we performed GO and KEGG enrichment analyses of the genes having positive and negative correlations with *PAICS*. GO enrichment revealed positively associated genes linked with catalytic activity, ribosome, mitochondria function, and RNA catabolism processes (Figure 5C). Alternatively, negatively correlated genes of *PAICS* were enriched with immune-related functions, immune-related cellular components, and immune-related biological processes (Figure 5C). Moreover, KEGG pathway enrichment supplemented the GO enrichment analyses results (Figure 5D). Furthermore, we performed correlation analysis of *PAICS* expression with immune cells and found that activated B cells, activated CD8^+^ T cells, and activated dendritic cells were negatively correlated with *PAICS* (Figures 5E–H). These findings explain that patients with high expression of *PAICS* have poor survival, which negatively correlated with the immune cells.

### Loss of Phosphoribosyl Aminoimidazole Succino Carboxamide Synthetase Inhibits Osteosarcoma Cell Proliferation and Migration and Induces Apoptosis

**FIGURE 6:**
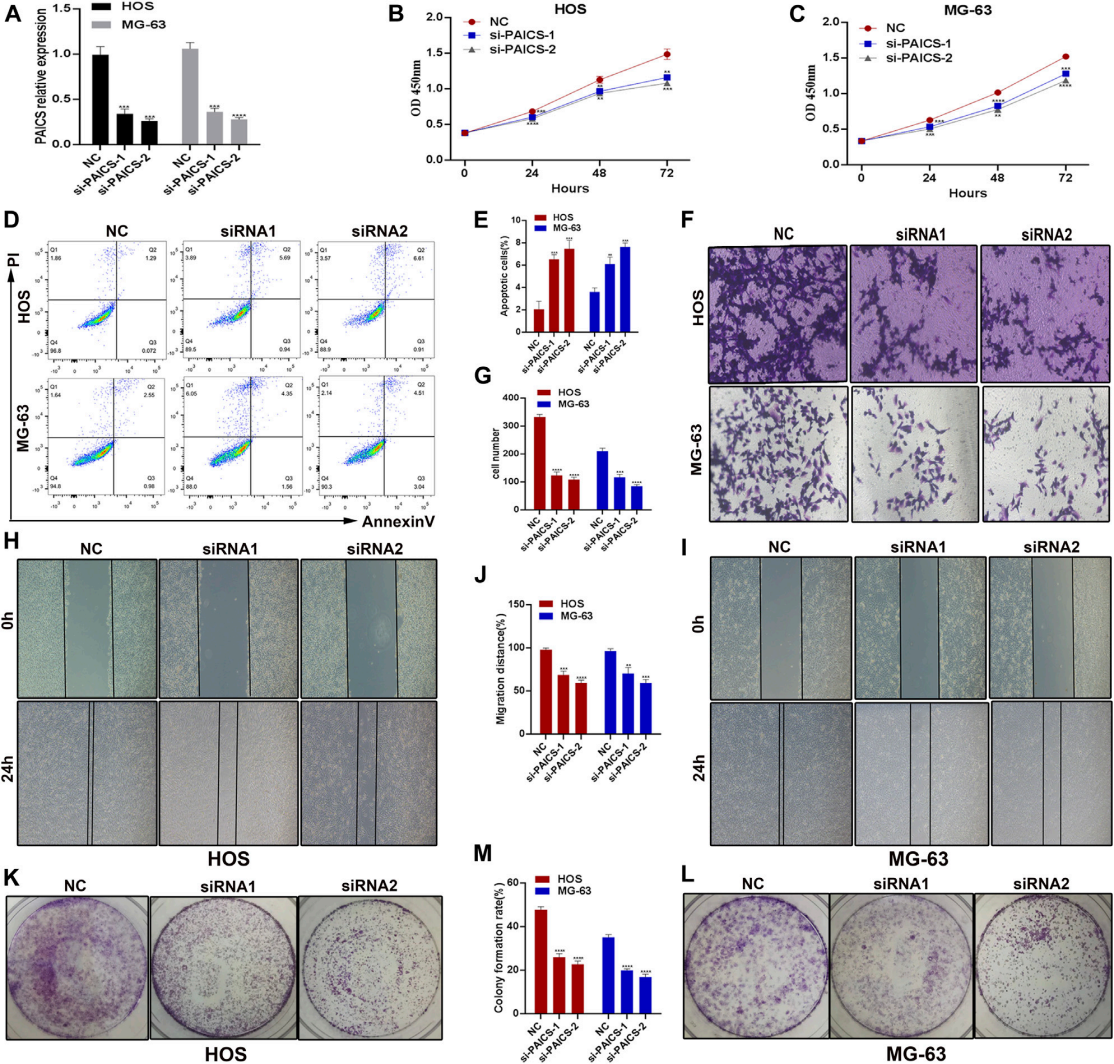
*PAICS* act as an oncogene. *PAICS* was silenced using siRNAs in two osteosarcoma cell lines HOS and MG-63 cells. **(A)** Knockdown efficiency of two siRNAs used to knockdown *PAICS*. **(B**, **C)** CCK8 assay showing reduced cell proliferation in knockdown cells. **(D**, **E)** Flow cytometry showing more apoptotic cells in KD cells, **(F**, **G)** Transwell assay showing reduced migration after siRNA-mediated silencing of *PAICS* in osteosarcoma cells. **(H**–**J)** Wound healing and **(K**–**M)** colony formation capability of the osteosarcoma cells were also reduced.

After establishing the fact that *PAICS* is associated with poor survival, we next determined the effect of the loss of *PIACS* on osteosarcoma cell lines HOS and MG-63. We first verified the knockdown of *PAIC* using different siRNAs in HOS and MG-63 cell lines. We found that si-PAICS-1 and si-PAICS-2 can successfully deplete the expression of *PAICS* to 60% and 70%, respectively (Figure 6A). Next, we performed CCK8 assay to determine the effect of *PAICS*’s silencing on cell proliferation. It was observed that depletion of *PAICS* had significantly reduced cell viability and proliferation in both cell lines HOS and MG-63 (Figures 6B,C). This prompted us to investigate the cell death upon knockdown of *PAICS*. Then, we subjected both cell lines HOS and MG-63 to flow cytometry analyses using annexin V/propidium iodide (PI) apoptosis staining kit. The data generated by flow cytofluorometry analyses showed a high population of apoptotic cells in si-*PAICS* HOS and MG-63 cells (Figure 6D). Then, we quantified these apoptotic cells’ population (6%–8%) and plotted them in a bar chart (Figure 6E). Furthermore, cell migration was also reduced in the knockdown cells, which was verified by Transwell cell migration assay (Figures 6F,G). Afterward, we performed a wound healing assay upon depleting *PAICS* in both cell lines HOS and MG-63 (Figures 6H,I). We found the loss of wound repair ability of cells by 20%–30% in si-*PAICS* cells (Figure 6J). Finally, we determined the ability of cells to form colonies upon knockdown of *PIACS* (Figures 6K,L), which discovered that *PAICS* loss in HOS and MG-63 cell lines also inhibited the capacity to form colonies (Figure 6M). All the above results conclude that *PAICS* promotes tumor growth by inhibiting apoptosis and inducing migration of the cells.

### Construction and Validation of Prediction Model

**FIGURE 7:**
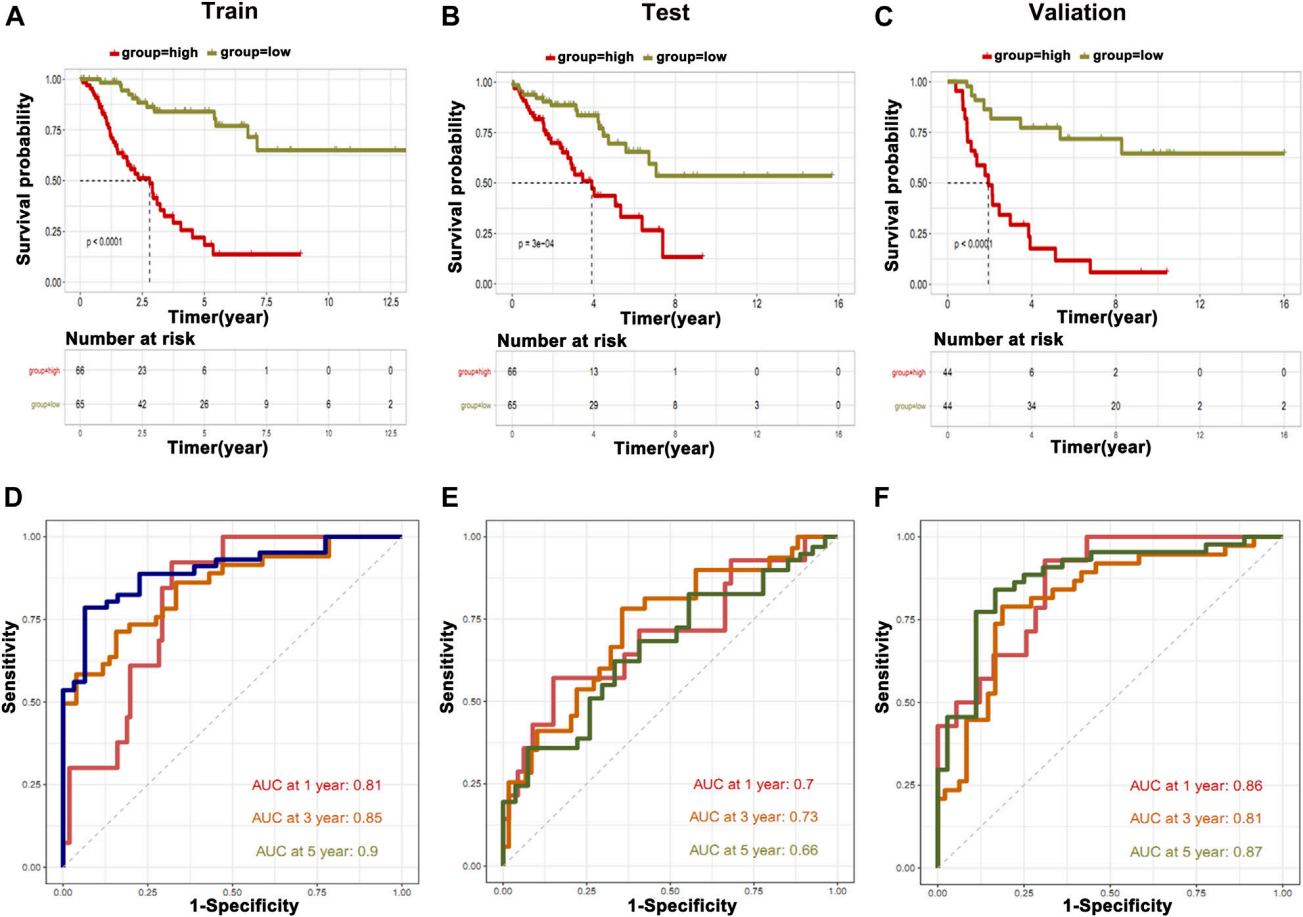
Construction of prediction model. The information of metabolic genes and their association with immune cells were used to construct and validate the prediction model. **(A)** Training cohort, **(B)** testing cohort, and **(C)** validation cohort. ROC curve analysis was applied on **(D)** training cohort, **(E)** testing cohort, and **(F)** validation cohort.

Based on the differential expression of the metabolic genes in clusters and their relationship to immune cells, we constructed and validated the prediction model by utilized metabolic genes. For this purpose, we devised two groups of low and highly expressed metabolic genes in osteosarcoma patients in the training, testing, and validation cohorts. We found that the high expression group showed poor OS than the low expression group (Figures 7A–C). To further validate our prediction model, we applied ROC curve analysis on training, testing, and validation cohorts, which revealed AUC at 1 year was 0.86 in the validation cohort, which showed strong performance (Figures 7D–F). Furthermore, partial likelihood deviance was observed in our prognostic model by Lasso Cox regression analysis (Supplementary Figure S5A). We found that eight hub genes could be potential prognostic factors (Supplementary Figure S5B). These findings may further contribute to the prognostic improvement of osteosarcoma patients.

## Discussion

Recently, the advancements and applications in bioinformatics tools made it easy to mine the potential gene signatures associated with the OS and prognosis of the cancers including osteosarcoma from publically available databases such as TCGA, TARGET, and GEO (Cao et al., 2020; Chen et al., 2020; Song et al., 2020; Wen et al., 2020; Xiao et al., 2020; Yang et al., 2021). Various signature genes have been found associated with the OS of osteosarcoma patients and tumor development. A recent study (Fan et al., 2021) identified the nine novel signature genes potentially predicting the OS and prognosis of osteosarcoma. Another study identified the three gene signature for successful prognostic prediction of osteosarcoma patients through GSEA (Yang et al., 2021). However, using a similar pattern, we identified novel potential metabolic gene signatures through comprehensive RNA-seq analyses in osteosarcoma tissues. In total, 876 genes related to metabolic function, OS, and prognosis of the osteosarcoma patients were identified. However, among them, the top 10 metabolic genes related to OS were prioritized of osteosarcoma patients. Of these top 10 genes, 3 metabolic genes (*ALDH1A1*, *HNMT*, and *NUDT7*), and 7 (*GL O 1*, *SLC19A1*, *SQLE*, *PAICS*, *PPAT*, *FASN*, *AK2*) were negatively and positively correlated with survival, respectively. We independently used these 10 gene signatures for training and validation cohort to construct Kaplan–Meier estimator, Cox proportional hazards model, and ROC curve and found significant association with OS and prognosis of the osteosarcoma.

It is a well-known fact that immune cell infiltration such as CD4^+^ naive T cells, CD4^+^ memory T cells, CD8^+^ T cells, CD8^+^ Tcm, B cells, and memory B cells in the TME are the key regulators of the tumor development and OS of the GC patients (Ren et al., 2021). Mast cells have also shown a clear association with the signature genes and prognosis of the cancer patients (Fan et al., 2021). Mast cells can expand the tumors by inducing angiogenesis and tissue remodeling, specifically changing the composition of the extracellular matrix, and also by promoting inflammatory pathways (Maciel et al., 2015). Mast cells also enhance the proliferation and migration of the dendritic cells, tumor-associated macrophages, and lymphocytes (Inagaki et al., 2016) and tissue homeostasis of TME, which facilitate the growth and progression of the tumors (Oldford and Marshall, 2015). It has also been confirmed that Mast cells may trigger some mechanisms that can affect the homeostasis of the osteosarcoma overall, affecting the occurrence and development of osteosarcoma (Campillo-Navarro et al., 2014; Maciel et al., 2015; Inagaki et al., 2016). In the current study, we analyzed the correlation between the infiltration of the immune cells, specifically activated CD8^+^ T cells and 876 differentially expressed metabolic genes, and found 206 differentially expressed metabolic genes were correlated to immune cells; specifically, the top three genes *PLAG2D*, *PIK3R5*, and *INPP5D* were positively associated, and *CTPS2*, *CACNB3*, and *ADCY6* were the top three negatively correlated metabolic genes with immune cells. Based on the immune scores, patients were classified into three clusters, C1, C2, and C3; patients in cluster 1 showed the lowest immune score as compared to other clusters; however, cluster 3 was found to have the highest immune score. In the next step, we determined the immune infiltration of the osteosarcoma clusters using the ssGSEA package and obtained 28 immune cells in osteosarcoma samples; as expected, cluster 3 was detected with a higher number of immune cells, such as monocytic lineage, T cells and CD8^+^ T. Previously, it has been suggested that most tumors at an advanced stage may have a higher frequency of mutations in genes related to the tumor immunity as compared to the early-stage tumors, which can activate more T cells and produce a stronger immune response (Ren et al., 2021). Usually, TME’s stromal cells express a large number of surface and secretory molecules, which directly inhibit CD4^+^ and CD8^+^ T cells, and activate immunosuppressed myeloid cells (Salmon et al., 2012; Turley et al., 2015). CD4^+^ T cells are the T helper cells, but also assist many other types of cells and act as a catalyst, increasing immune protection through many different pathways (Jaigirdar and Macleod, 2015).

Furthermore, we analyzed the expression of genes in all clusters showing that cluster 3 specifically has a high expression of the genes that are found downregulated in other clusters; thus, these genes were associated with immune function. Among all genes in three clusters, *PAICS* was commonly expressed in all clusters; therefore, we chose it for further validations. *PAICS* is an essential enzyme that has a significant role in *de novo* purine biosynthesis and associated with the formation of various tumors (Huang et al., 2020). *PAICS* has been localized in lung cancer tissues and shows high expression in tumor tissues as compared to normal tissues. High expression of *PAICS* has also been associated with the poor prognosis of lung cancer and gastric cancer patients (Goswami et al., 2015; Huang et al., 2020). Mechanistically, *PAICS* regulates pyruvate kinase activity, cell proliferation, and invasion (Goswami et al., 2015). Furthermore, its high expression has also been associated with the aggressiveness of the prostate cancer; therefore, *PAICS* has been considered essential for the proliferation and invasion of prostate cancer cells (Chakravarthi et al., 2018). Silencing its expression in breast cancer cells significantly reduced cell viability and proliferation (Meng et al., 2018). Increased expression of *PAICS* is involved in the proliferation, migration, invasion, and growth of colorectal cancer (CRC) cells, while depleting *PAICS* in the mice reduced tumor growth and metastasis to the liver, lung, and bones (Agarwal et al., 2020).

We performed survival analysis for the patients with low and high expression of *PAICS*. Interestingly, we observed that patients with high expression of *PAICS* had a poor survival, and *vice versa*. Furthermore, the expression of *PAICS* was negatively correlated with the immune cells, specifically with activated B cells, activated CD8^+^ T cells, and activated dendritic cells, thus proving that patients with high expression of *PAICS* have poor survival. *PAICS* was highly expressed in 70% of the CRC tissues and associated with poor 5-year survival of the patients regardless of the pathological stages, patients’ race, gender, and age (Agarwal et al., 2020). Based on current results, knockdown of *PAICS* in osteosarcoma cells (HOS and MG-63) significantly reduced the cell proliferation, migration, and wound healing ability and induced apoptosis, which revealed the oncogenic role of *PAICS* in the osteosarcoma. Our current research has some limitations; for example, the gene signature still needs to be verified for its clinical use. Accordingly, further studies are required to uncover the relationships between the gene signature and osteosarcoma progression.

## Conclusion

In summary, the genetic or physiological alterations affect the regulation of metabolic gene signatures in TME, leading to cancer development; however, it has not fully been explained in osteosarcoma. We observed the significant difference of metabolic genes’ expression between osteosarcoma and normal samples. Our analyses identified the correlation of a list of metabolic genes with OS and immune cell infiltration in osteosarcoma patients and TME. Through these analyses, we identified *PAICS* as a potential candidate gene for further analyses as it showed association with poor survival and immune cells. Furthermore, we established that loss of *PAICS* induces apoptosis and inhibits the proliferation and migration of HOS and MG-63 cell lines. Thus, we conclude that *PAICS* acts as an oncogene in osteosarcoma and could be used as a potential diagnostic and prognostic marker.

## Data Availability

The original contributions presented in the study are included in the article/Supplementary Material, further inquiries can be directed to the corresponding author.
