# Supplementary figures and images for: Global Characterization of Metabolic Genes Regulating Survival and Immune Infiltration in Osteosarcoma
Source: Front Genet. 2022 Jan 13;12:814843. doi: 10.3389/fgene.2021.814843 (PMC8793845; doi:10.3389/fgene.2021.814843)

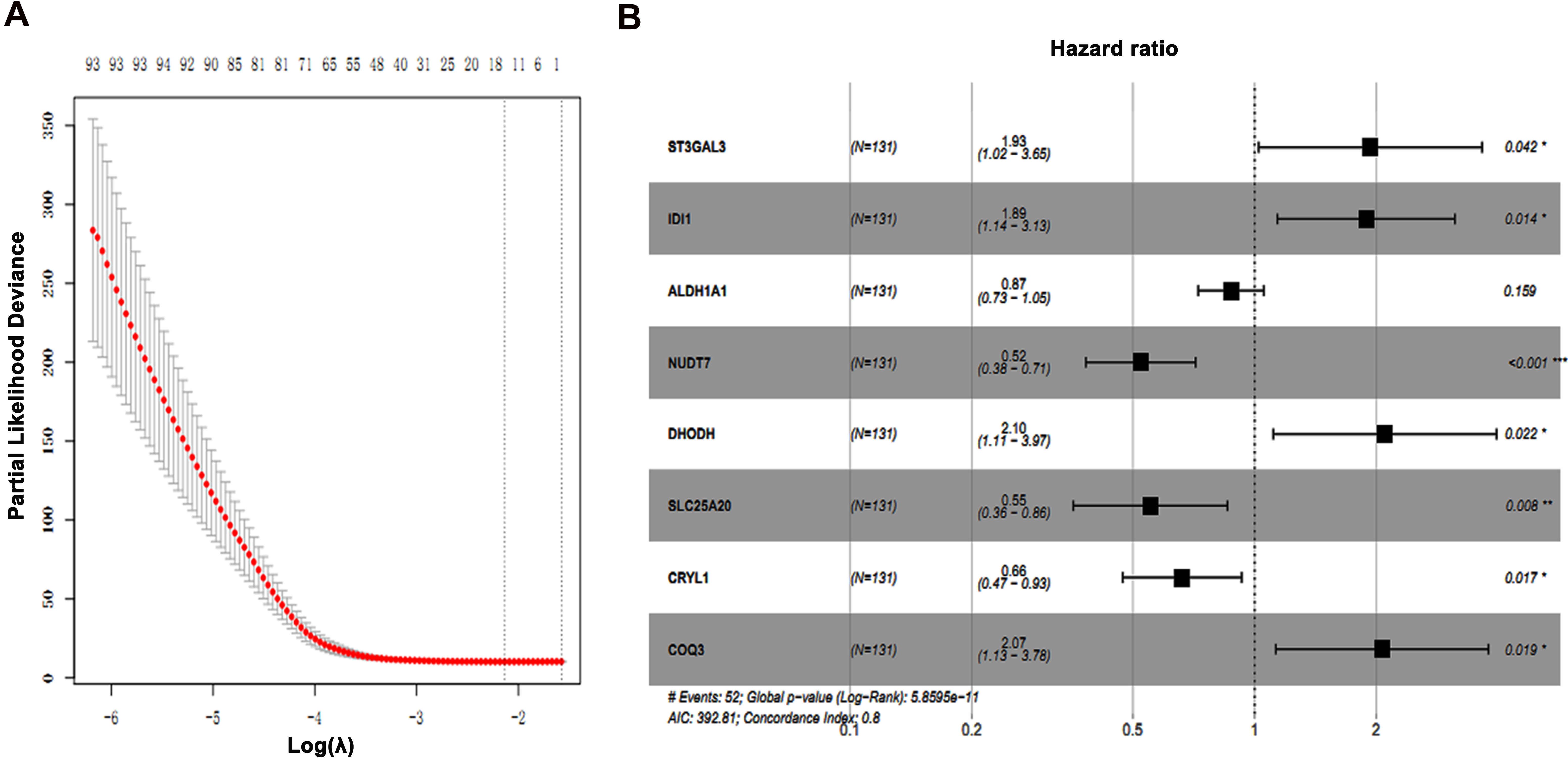

Supplement: Supplementary file 1 [file Image5.jpg]

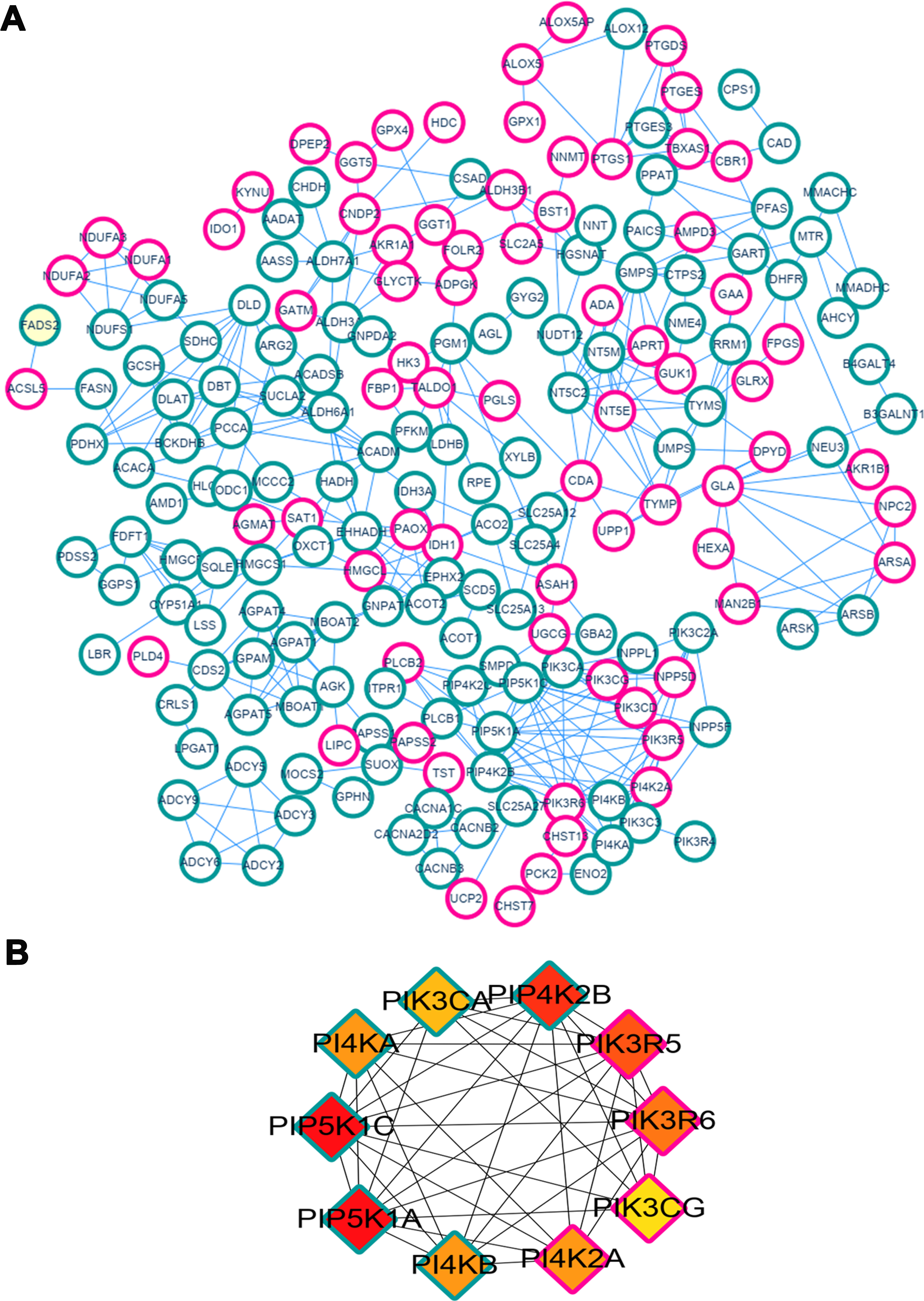

Supplement: Supplementary file 2 [file Image3.jpg]

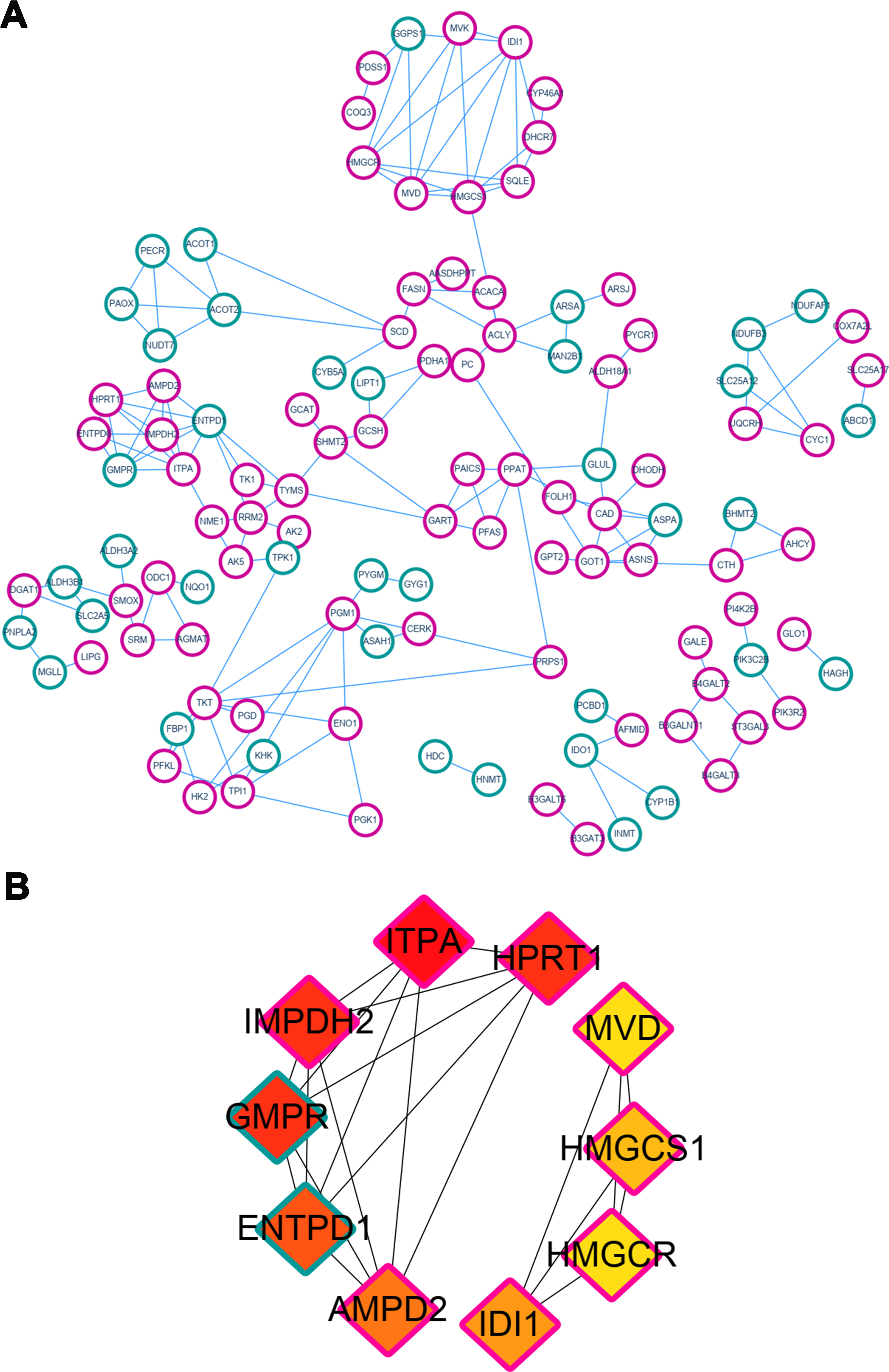

Supplement: Supplementary file 3 [file Image2.jpg]

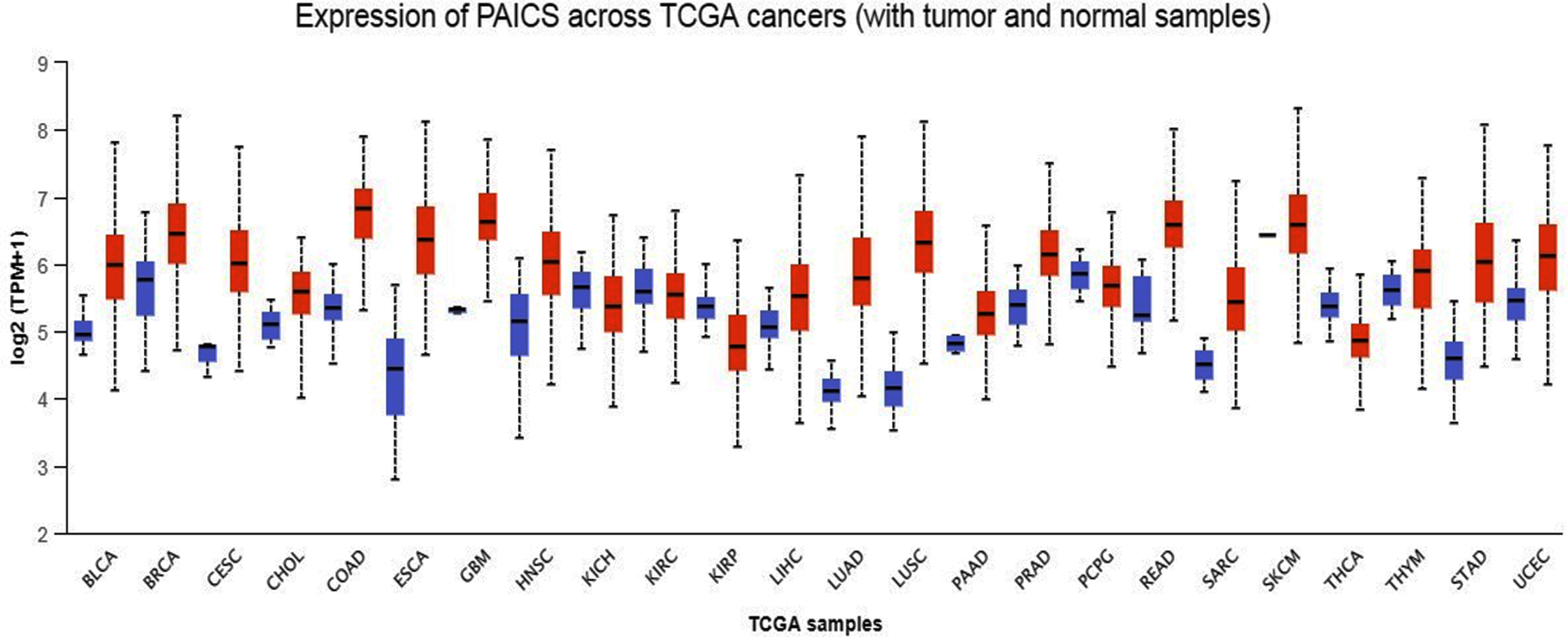

Supplement: Supplementary file 5 [file Image4.jpg]

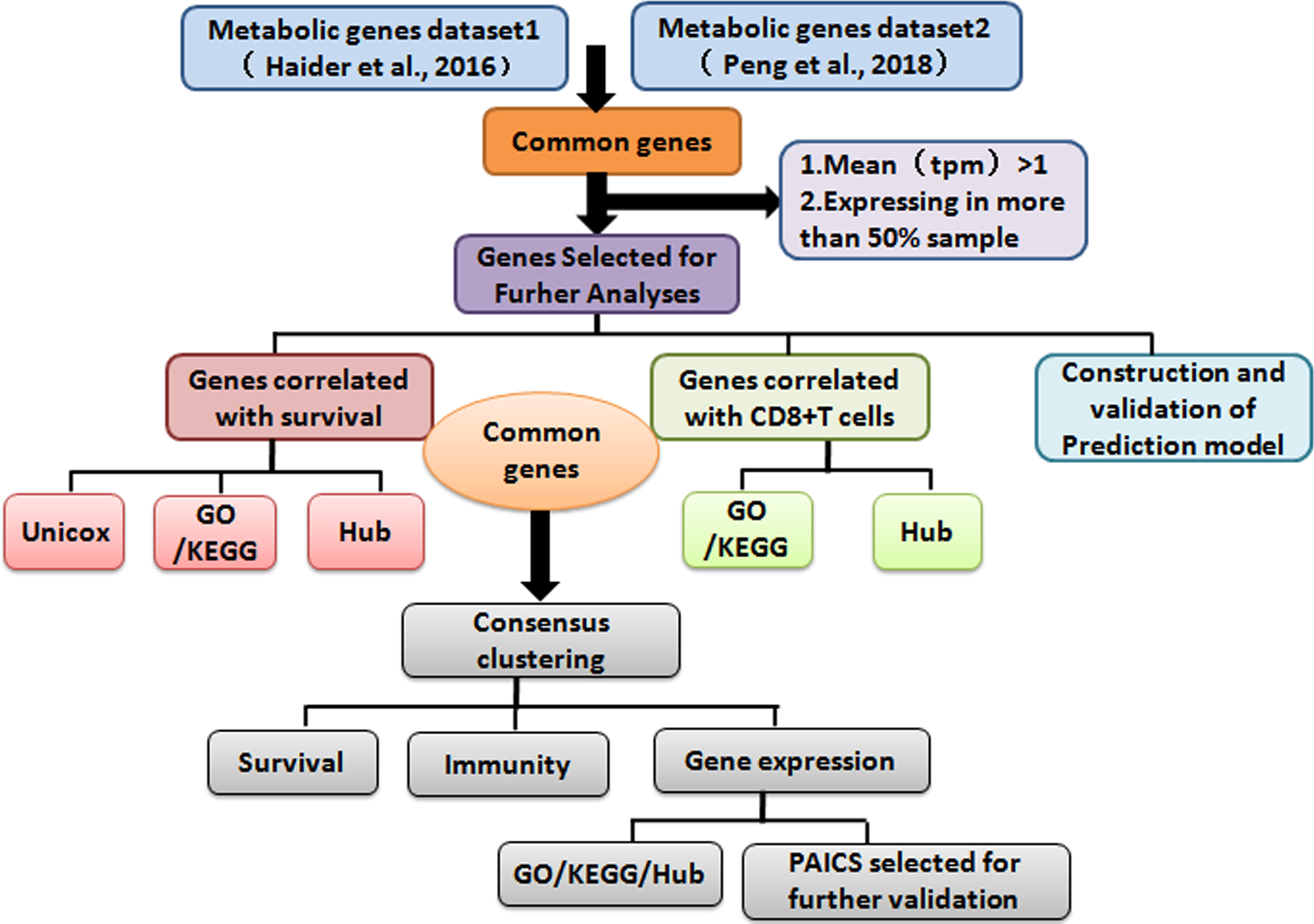

Supplement: Supplementary file 6 [file Image1.jpg]
