# Supplementary material for: Global Characterization of Metabolic Genes Regulating Survival and Immune Infiltration in Osteosarcoma
Source: Front Genet. 2022 Jan 13;12:814843. doi: 10.3389/fgene.2021.814843 (PMC8793845; doi:10.3389/fgene.2021.814843)
Supplement: Supplementary file 4 [file Table1.DOC]

**Supplementary Table 1: Primers used in Real-time quantitative PCR**

analysis and the small RNA interfering assay.

| Name | Sequence (5′-3′) |
| --- | --- |
| PAICSF | GCAGGGTTGTAGTGTTGATGG |
| PAICSR | GCCACTGCCACAAATACAGTAG |
| GAPDHF | AGAAGGCTGGGGCTCATTTG |
| GAPDHR | AGGGGCCATCCACAGTCTTC |
| PAICS-SiRNA1F | GGGCUCCAAAUGGUAAAGATT |
| PAICS-SiRNA1R | UCUUUACCAUUUGGAGCCCTT |
| PAICS-SiRNA2F | GCAGGGUUGUAGUGUUGAUTT |
| PAICS-SiRNA2R | AUCAACACUACAACCCUGCTT |
